# Metabolomic Analysis Reveals Nutritional Diversity among Three Staple Crops and Three Fruits

**DOI:** 10.3390/foods11040550

**Published:** 2022-02-15

**Authors:** Yunxia Shi, Yanxiu Guo, Yuhui Wang, Mingyang Li, Kang Li, Xianqing Liu, Chuanying Fang, Jie Luo

**Affiliations:** 1 School of Tropical Crops, Hainan University, Haikou 570288, China; yunxiashi225@163.com (Y.S.); gyx13563502250@163.com (Y.G.); wyh0140@163.com (Y.W.); lygaugau@163.com (M.L.); kang_li@hainanu.edu.cn (K.L.); liuxq@hainanu.edu.cn (X.L.); cyfang@hainanu.edu.cn (C.F.); 2 Hainan Yazhou Bay Seed Laboratory, Sanya Nanfan Research Institute of Hainan University, Sanya 572025, China

**Keywords:** crops, fruits, nutrition, metabolite analyses, metabolome

## Abstract

More than 2 billion people worldwide are under threat of nutritional deficiency. Thus, an in-depth comprehension of the nutritional composition of staple crops and popular fruits is essential for health. Herein, we performed LC-MS-based non-targeted and targeted metabolome analyses with crops (including wheat, rice, and corn) and fruits (including grape, banana, and mango). We detected a total of 2631 compounds by using non-targeted strategy and identified more than 260 nutrients. Our work discovered species-dependent accumulation of common present nutrients in crops and fruits. Although rice and wheat lack vitamins and amino acids, sweet corn was rich in most amino acids and vitamins. Among the three fruits, mango had more vitamins and amino acids than grape and banana. Grape and banana provided sufficient 5-methyltetrahydrofolate and vitamin B6, respectively. Moreover, rice and grape had a high content of flavonoids. In addition, the three crops contained more lipids than fruits. Furthermore, we also identified species-specific metabolites. The crops yielded 11 specific metabolites, including flavonoids, lipids, and others. Meanwhile, most fruit-specific nutrients were flavonoids. Our work discovered the complementary pattern of essential nutrients in crops and fruits, which provides metabolomic evidence for a healthy diet.

## 1. Introduction

A healthy diet containing enough nutrients is vital to health. The lack of macro- and micro-nutrients in diets threatens health [1], especially in underdeveloped countries where people cannot afford varied diets [2,3]. Rice, wheat, and corn are globally major staple crops. Although they provide 60% of the world’s calorie intake [4], the micro-nutrients available from staple foods are limited. For instance, corn lacks tryptophan and lysine, two essential amino acids for humans [5]. The processing of rice and wheat removes bran and embryo, which leads to the loss of most nutrients [6,7].

The benefits of a plant-based diet on health are mainly from the composition of phytochemicals [8,9]. More fruit uptake can reduce the risk of some diseases for the enrichment of vitamins and other micronutrients [10,11]. Mango, banana, and grape are globally the most widely cultivated fruits [12,13]. Mango is rich in phytosterols, carotenoids, and vitamins [14,15]. Phytochemicals in mango protect humans from diabetes, obesity, and cancer [16]. Banana contains high levels of provitamin A carotenoids (pVACs), which alleviate vitamin A deficiency and reduce the risk of cancers and heart diseases [17]. As a popular fruit, grape is famous for highly bioactive phenols, such as anthocyanins, flavanols, flavonols, and resveratrol. These compounds are pivotal in antioxidant, heart protection, anti-cancer, and anti-aging activity [18,19]. Therefore, through food selection and a healthy diet, people can get enough nutrition and reduce the occurrence of nutritional deficiency. However, metabolic cues for food selection are limited.

Metabolomic studies provide novel insights into the composition of phytochemicals. Metabolic diversity among staple food crops has been well documented. For instance, metabolome signatures have varied across developmental stages and genotypes in rice, maize, and wheat [20,21,22]. Multiple-omics studies have also provided deep insights into genetic bases of metabolic diversity in staple crops [23,24,25]. Recently, metabolic features of fruits have attracted more and more interest. For example, citrus fruits are primary fruit sources of poly methoxy flavonoids (PMFs), and the determination of major PMFs in fruits or leaves of 116 citrus accessions revealed significant species-specific and spatiotemporal characteristics. All reticulated citrus and its natural or artificial hybrids had detectable PMFs, especially in fruits of wild or early cultivated citrus in early fruit development [26]. In peach, metabolomic analysis was used to construct the metabolic network of peach mesocarp throughout development. In the early developmental stages of peach, protein abundance was significantly reduced, while bioactive polyphenols and amino acids piled up [27]. However, the diversity of nutritional metabolites between crops and fruits is largely unknown.

Here, we performed non-targeted and targeted metabolic profiling to dissect the metabolic diversity by using rice, wheat, corn, banana, grape, and mango. We detected over 3000 metabolites. Those include amino acids, vitamins, flavonoids, lipids, and other nutrients. In addition, we found significant differences in nutrient construction between crops and fruits. Although crops are rich in lipids and sweet corn is rich in vitamins and amino acids, rice and wheat lack in vitamins and amino acids. Grape is rich in flavonols and anthocyanin, but it lacks amino acids. Mango is rich in vitamins, especially vitamin C, and most of amino acids. Banana is rich in vitamin B6 but short in flavonoids. Based on the rich metabolic diversity between crops and fruits, our work deepens our comprehension of the nutrient structure of a diet.

## 2. Materials and Methods

### 2.1. Plant Materials

To study the difference in metabolites between crops and fruits, we selected three important crops and three popular fruits for research. Crops included wheat, corn, and rice. Fruits included grape, mango, and banana. Rice (ZH11) came from the breeding base of Hainan University. Corn (sweet corn) came from Ding’an breeding base of Hainan University. Wheat (Chinese Spring) came from common wheat varieties grown in the experimental station of the Institute of genetics and developmental biology, Chinese Academy of Sciences (IGDB, CAS) from 2018 to 2019. Grapes (Pinor Vermei, one of the most popular grape varieties in the world), mangoes (Alphonso, a traditional Indian cultivar) and bananas (Cavendish) were selected from the field germplasm community of Guangzhou banana garden.

### 2.2. Chemical Reagents

Chromatographic-grade acetonitrile, acetic acid, and methanol were purchased from Merck (Darmstadt, Germany). The Milli-Q water was purified using a Millipore purification system (Millipore Corporation, Burlington, MA, USA). All standards used in the test were stored in −80 °C refrigerator in the dark.

### 2.3. Metabolite Sample Preparation

Fresh fruit or dry grain of crop were collected into 50 mL centrifuge tubes and quickly frozen in liquid nitrogen and freeze dried. Three biological replicates were collected for each species. The samples were ground into powder using a grinder machine (MM400, Retsch) with steel balls at 28 Hz for 56 s or more. Then, 0.05–0.1 g of sample powder was suspended with 70% methanol water solution in the ratio of 1:10,000. Next, the samples were extracted by ultrasonic wave for 10 min at 50 Hz for a total of three times [22,28]. At the end of each time, vortex vibration and mixing were required.

### 2.4. Metabolomic Detection

Non-targeted metabolic profiling analyses were performed with Q Exactive Focus Orbitrap LC-MS/MS (Thermo Scientific, Waltham, MA, USA). Scanning mass ranged from *m*/*z* 100–1000 with an accumulation time of 0.10 s. The scanning mode was full MS/ddMS2. The recorded data were processed with compound discoverer (CD) 3.1 software to obtain the mass to charge ratio, retention time, MS/MS2 information of all detected substances. Then, the detected signals were automatically matched through the internally established reference libraries of chemical standard entries of software to predict and identify the metabolite information. The multiple reaction monitoring (MRM) mode with QTRAP 6500^+^ LC-MS/MS (Shimadzu, Kyoto, Japan) was used for targeted metabolome analyses. The detection window was set to 80 s, and the targeted scanning time was 1.5 s. The original data were processed by Multi Quant 3.0.3 software. The chromatographic column was C18 column (Shim-pack GLSS C18, 1.9UM, 2.1*100, Shimadzu). Mobile phase A and B was 0.04% acetic acid–water solution, and mobile phase B was 0.04% acetic acid–methanol solution. The qualitative and quantitative chromatographic conditions were consistent.

### 2.5. Statistical Analysis

The relative signal strength of metabolites was divided and normalized according to the internal standard (0.1 mg L^−1^ lidocaine), and log2 was then used to transform the value. We used Student’s t-test and fold change of difference to screen for differentially accumulated metabolites (DAMs). Metabolites with *p* < 0.05 and |log2 (fold change) |≥ 1 were considered as DAMs. The differences between the metabolites in six fruits were calculated by nested ANOVA in the R package.

## 3. Results

### 3.1. Metabolic Analysis of Crops and Fruits

**Figure 1:**
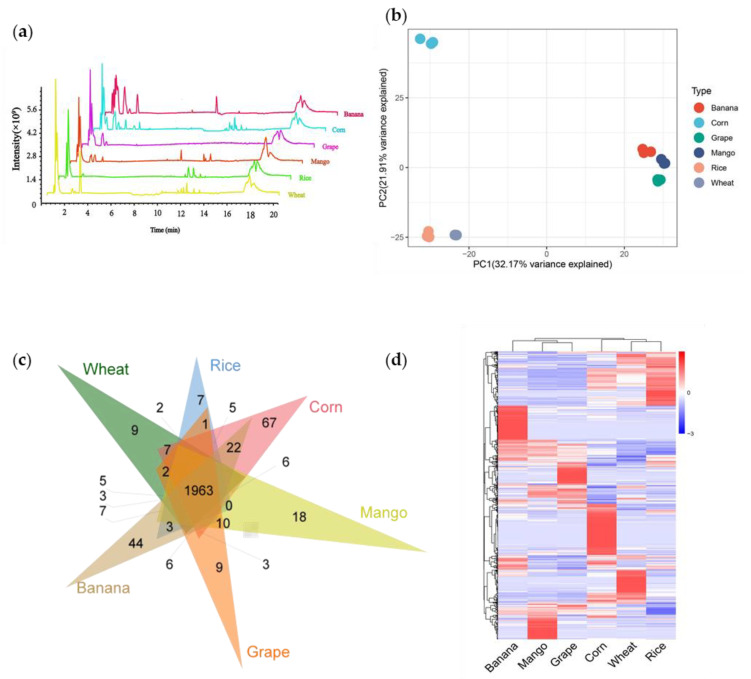
Analysis of metabolic variation in fruits and crops using Q Exactive Focus Orbitrap LC-MS/MS. (**a**) Total ion chromatography of metabolites in fruits and crops. (**b**) Principal component analysis (PCA) of the total ion chromatography of fruits and crops. (**c**) Venn diagram analysis of crops and fruits. (**d**) Heat map analysis of 2631 metabolites detected in crops and fruits.

To dissect the diversity of metabolites between crops and fruits, we selected three staple food crops (rice, corn, and wheat) and three popular fruits (mango, grape, and banana). Through non-targeted metabolome detection, we detected a total of 13,790 metabolic signals in six species (Figure 1a). Among them, 7831 signals were detected in rice, 8325 signals in wheat, 8879 signals in corn, 8074 signals in mango, 8135 signals in grape, and 9139 signals in banana (Table S1). Next, we performed a principal component analysis (PCA) of all samples based on the liquid chromatography-mass spectrometry (LC-MS) data. PCA diagram showed that principal component (PC) 1 and 2 explained 32.17% and 21.9% variability, respectively (Figure 1b). Principal component 1 separated crops and fruits successfully, indicating that the diversity of metabolites between crops and fruits was significant.

To further study the metabolic feature of crops and fruits, we quantified the metabolites by scheduled multiple reaction monitoring (SMRM) and finally detected 2631 metabolites. We detected 2296, 2251, 2432, 2290, 2249, and 2376 metabolites in wheat, rice, corn, mango, grape, and banana, respectively (Table S2). Among them, 1963 metabolites were shared by crops and fruits. Meanwhile, 9, 7, 67, 18, 9, and 44 metabolites only existed in wheat, rice, corn, mango, grape, and banana, respectively (Figure 1c). The distribution of the metabolites of crops and fruits is complementary. Substances with high content in crops are relatively low in fruits, and vice versa (Figure 1d).

### 3.2. Characterization of Metabolic Signals

**Figure 2:**
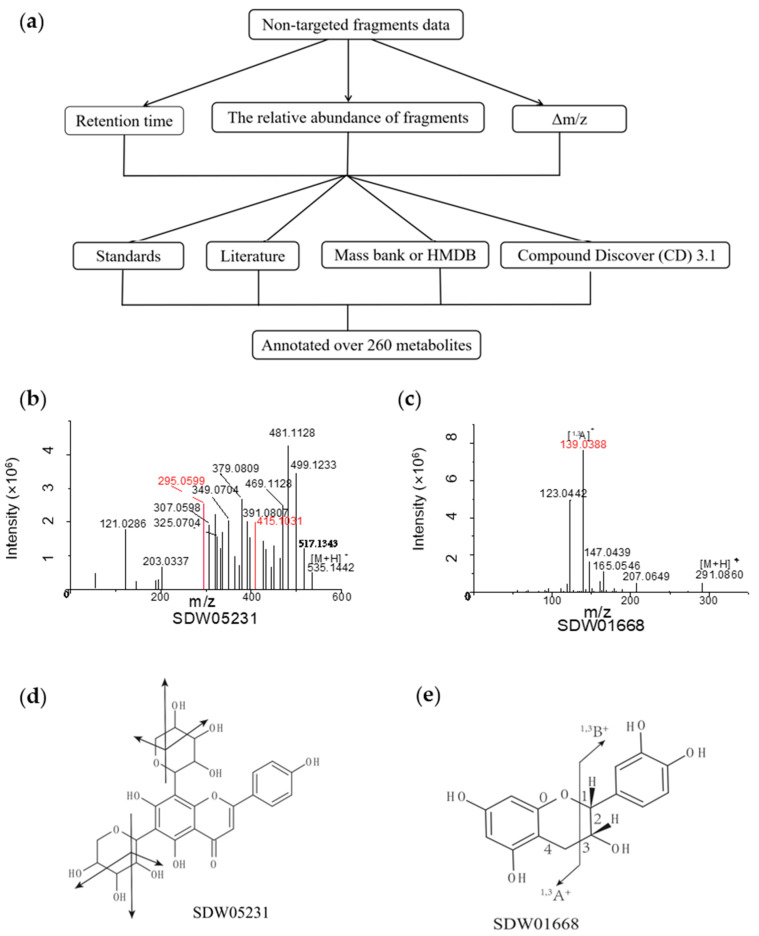
Detection and identification of specific metabolite signs by Q Exactive Focus Orbitrap LC-MS/MS. (**a**) Flowchart for detection and identification of specific metabolites. (**b**) MS/MS spectra of SDW05231 at *m*/*z* 535.1442, the metabolite was identified as 5,7-dihydroxy-2-(4-hydroxyphenyl)-6,8-bis(3,4,5-trihydroxyoxan-2-yl)-4H-chromen-4-one. (**c**) MS/MS spectra of SDW01668 at *m*/*z* 291.0860, the metabolite was identified as (-)-Epicatechin. (**d**) The molecular structure of the 5,7-dihydroxy-2-(4-hydroxyphenyl)-6,8-bis(3,4,5-trihydroxyoxan-2-yl)-4H-chromen-4-one and its general fragmentation rules. (**e**) The molecular structure of the (-)-Epicatechin and its general fragmentation rules.

We characterized species-specific metabolites by the retention time, the relative abundance of fragments, and the mass loss during fragmentation. Then, according to these characteristics, we checked the fragment information in literature and databases, such as mass bank [29] and the Human Metabolome Database (HMDB) [30]. Finally, we annotated some of the metabolites with the help of standards. At the same time, by using Compound Discoverer (CD) 3.1, we matched metabolic structures through the CD database (Figure 2a).

The crop-specific SDW05231 (RT 6.26 min) yielded a precursor ion [M+H]^+^ at *m*/*z* 535.1442. We observed product ions with uneven abundance in the secondary mass spectrum. The precursor ion lost 18 (H_2_O) or 30 (CH_2_O) and produced two signals at *m*/*z* 415.1031 [M+H-120]^+^ and *m*/*z* 295.0599 [M+H-120]^+^ (red peak spectrum in Figure 2b). Its fragmentation pattern resembled the flavone C-glucoside, which contains two pentose residues. Considering the product ion at *m*/*z* 295.0599, we inferred that SDW05231 was an apigenin derivative. Therefore, we decoded DWZP05231 as 5,7-dihydroxy-2-(4-hydroxyphenyl)-6,8-bis(3,4,5-trihydroxyoxan-2-yl)-4H-chromen-4-one (Figure 2b,d).

Then, we annotated a fruit-specific SDW01668 as (-)-Epicatechin using the CD database. SDW01668 (RT 4.73 min) produced a precursor ion [M+H]^+^ at *m*/*z* 291.0860. The tandem mass spectrum showed a high-intensity fragment [1,3A]^+^ ion at *m*/*z* 139.0388. A further loss of 16 Da yielded *m*/*z* 123.0442 based on [1,3A]^+^. The secondary fragments’ *m*/*z* and fracture modes were highly similar to (-)-Epicatechin (Figure 2c,e).

We annotated over 260 metabolites (Table S3), 28 and 18 of which were absent in fruits and crops, respectively. Moreover, we also identified species-specific metabolites, such as resveratrol in grapes (Table S4).

### 3.3. Whole Metabolome Scale Comparative Analysis of Crops and Fruits

**Figure 3:**
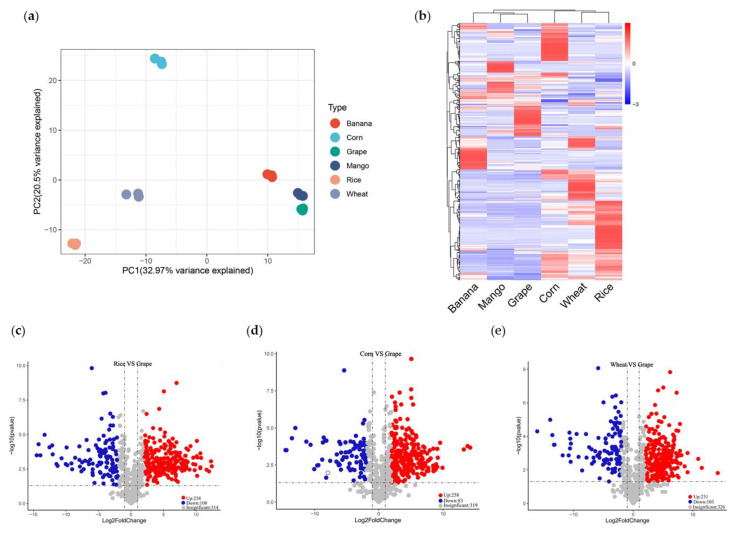
Q trap 6500^+^ LC-MS/MS was used to analyze metabolite changes in crops and fruits. (**a**) Principal component analysis of 660 metabolites detected in crops and fruits. (**b**) Heat map analysis of 664 metabolites detected in crops and fruits. (**c**) Volcanic map analysis of differentially accumulated metabolites in rice and grape. (**d**) Volcanic map analysis of differentially accumulated metabolites in corn and grape. (**e**) Volcanic map analysis of differentially accumulated metabolites in wheat and grape. The average of three biological replicates was used for metabolite analysis. The content of each metabolite was normalized, and hierarchical clustering was carried out. Each crop and fruit was labeled in a single column, and each metabolite was represented by a single row.

To explore the diversity of nutrients between crops and fruits, we performed LC-MS/MS-based targeted metabolome analyses. Firstly, we quantified the metabolites by scheduled multiple reaction monitoring (SMRM). We detected 660 metabolites, including 26 vitamins, 106 amino acids and derivatives, 225 lipids, 108 flavonoids, 73 organic acids, and other metabolites (Table S5). A PCA showed that PC1 and PC2 explained 32.97% and 20.5% of the variability, respectively (Figure 3a). PC1 separated crops and fruits, indicating the significant metabolic difference between them. According to PC2, grape, mango, rice, and wheat clustered together. Meanwhile, banana and corn got close to each other and were far away from the others. The species-dependent accumulation pattern was further visualized by a heatmap based on crop and fruit metabolome data. The six species formed two clusters: crops and fruits (Figure 3b). That is, the metabolic features of crops and fruits differed remarkably.

Then, we analyzed differentially accumulated metabolites (DAMs) between each fruit with three crop species. DAMs between each species pair met the following criterion: the fold change >2, while the *p*-value <0.05. Grapes, for example, accumulated 341~346 DAMs compared with crops. A total of 346 DAMs existed between rice and grape, including 35 amino acids and derivatives, 70 flavonoids, 141 lipids, 15 vitamins, and others. Rice harbored 238 up-regulated and 108 down-regulated metabolites (Figure 3c). We found 341 DAMs between corn and grape, consisting of 52 amino acids and derivatives, 66 flavonoids, 118 lipids, 22 vitamins, and others. Compared with those in grape, 258 and 83 metabolites accumulated with elevated and reduced levels in corn (Figure 3d). The 334 DAMs between wheat and grapes included 41 amino acids and derivatives, 70 flavonoids, 129 lipids, 15 vitamins, and others. Compared with grapes, wheat produced 231 and 103 metabolites with significantly higher and lower levels, respectively (Figure 3e). Compared with crops, DAMs of mango and banana were mainly amino acids and derivatives, flavonoids, lipids, and vitamins (Figure S1).

### 3.4. Comparative Analysis of Common Existing Nutrients in Crops and Fruits

**Figure 4:**
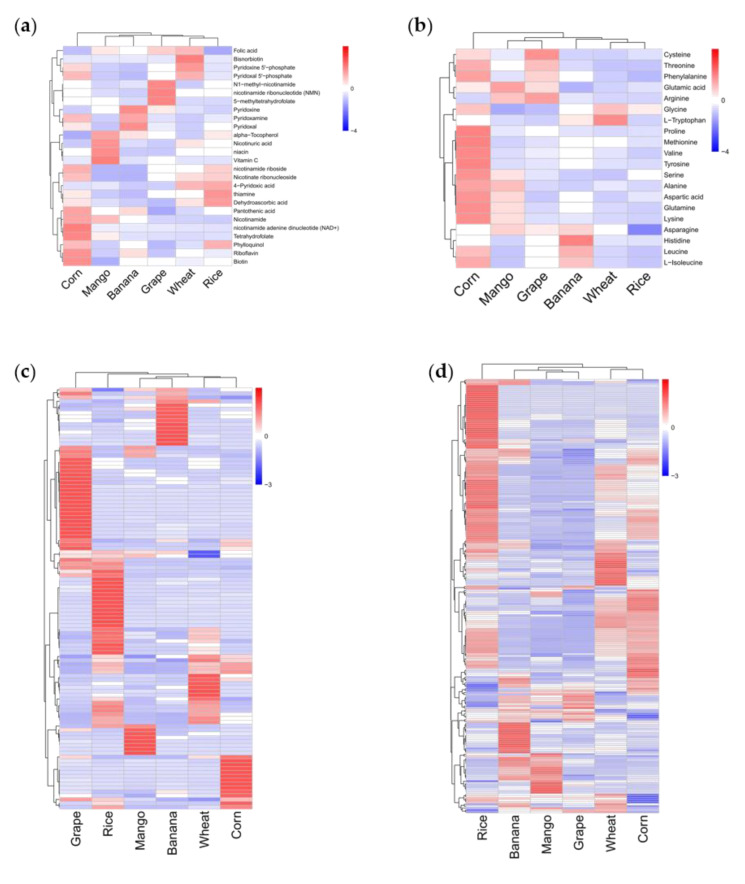
Accumulation of vitamins and amino acids in three crops and three fruits. (**a**) Heat map of vitamin in three crops and three fruits. (**b**) Heat map of amino acid in three crops and three fruits. (**c**) Heat map of flavonoids in three crops and three fruits. (**d**) Heat map of lipids in three crops and three fruits. The average of three biological replicates was used for metabolite analysis. The content of each metabolite was normalized, and hierarchical clustering was carried out. Each crop and fruit is marked in a single column, and each metabolite is represented by a single row.

To comprehend the nutritional value of crops and fruits, we firstly focused on common existing nutrients. The accumulation patterns of vitamins are complementary in crops and fruits. Corn was rich in most B vitamins, including tetrahydrofolate, nicotinamide, biotin, pyridoxal, pyridoxamine, and riboflavin. Rice and wheat accumulated the most vitamin B1 and phosphorylated B6, respectively (Figure 4a). Fruits contained vital compounds limited in crops. For instance, mango produced more vitamin C, and grape provided high content of 5-methyltetrahydrofolate. These vitamins are cofactors of many enzymes. Banana yielded a high level of non-phosphorylated vitamin B6 (Figure 4a). To conclude, the categories of vitamins between crops and fruits were significantly different. Crops were rich in B vitamins, which may exist in bran and corn endosperm. Fruits supplemented other necessary vitamins, such as vitamin C.

The accumulation of amino acids was also different in crops and fruits. Corn yielded high levels of essential amino acids, excluding tryptophan, while wheat produced considerable content of tryptophan. Compared with grapes and banana, mango contains more amino acids (Figure 4b).

Lipids and flavonoids also differed in crops and fruits. Flavonoids are highly accumulated in grapes and rice, but less so in the other species (Figure 4c). The accumulation patterns across species were complementary. Flavanones and anthocyanins mainly existed in grapes, while flavones were mainly present in rice. Crops produced more lipids than fruits, especially more than grape (Figure 4d).

### 3.5. Comparative Analysis of Species-Specific Nutrients

Some nutrients showed remarkable species dependence. We focused on compounds commonly present in all fruits but absent in crops. We detected five fruit-specific flavonoids, including (-)-epigallocatechin, catechin, (+)-gallocatechin, (-)-gallatechin, and eriodictyol 7-O-glucoside. Meanwhile, crops harbored more kinds of specific metabolites, consisting of four flavonoids, three lipids, and four others (Table 1). Then, we noticed metabolites existing in a single species: One in wheat, twelve in rice, five in corn, five in mango, five in grape, six in banana (Table S5). In addition, we analyzed some crucial metabolites, such as chlorogenic acid and theanine. Chlorogenic acid was detected only in corn, grape, and banana. Although theanine was present in all species, it accumulated at higher levels in mango and grape (Table S5).

## 4. Discussion

Crops and fruits are indispensable for humans. They provide a variety of nutrients and protect humans against diseases. Hence, nutritional metabolomics of crops and fruits are essential to dissect the nutritional value and maintain a healthy diet. Although metabolome studies have been conducted in crops and fruits, metabolic differences between crops and fruits are yet to be drawn. In this study, we detected 2631 and 660 compounds in crops and fruits using non-targeted and targeted LC-MS, respectively. Metabolic features differed remarkably in crops and fruits. Moreover, this work revealed the complementary pattern of nutrient accumulation in different species, which provides metabolic insights into food choice.

Plant metabolites play vital roles in plant growth and nutrition [31]. Tremendous metabolic diversity occurs across different species [32]. An investigation on metabolomic data has illustrated interspecific metabolic variation in rice and maize populations, and identified flavonoids and phenolamides as contributory compounds in diversified evolution in rice and maize [33]. A study with widely targeted LC-MS/MS has analyzed metabolic features of the pearling fractions and discovered the enrichment of health-beneficial metabolites in the wheat bran. Meanwhile, the authors have also found that flavonoids are of the most remarkable spatial divergence among grain layers [34]. In ten fruits, the diversity of more than 2000 metabolites has been studied and used to construct a metabolic evolutionary tree [32]. Through the analysis of metabolites in the fruits and leaves of 12 kinds of piper, it was found that, although there were significant differences in chemical richness and structural complexity between different species, fruit diversity exceeded leaf diversity [35]. Although these works have elucidated some of the metabolic diversity in different species, our work directly provides metabolic evidence for the necessity of a balance between staple foods and fruit uptake. In this study, widely targeted metabolomics were used to study the metabolic diversity in major food crops (rice, wheat, and corn) and three fruits (mango, grape, and banana). The main differential metabolites in crops and fruits were vitamins, amino acids, flavonoids, and lipids. They are essential in plant growth and development, as well as in keeping humans fit.

Common existing nutrients displayed remarkable species-dependent accumulation [36,37]. Independent work has documented accumulation patterns of flavonoids in rice and grape, respectively [33,38]. By using a comparative metabolomic analysis, we found that flavonoids were most abundant in rice and grape among the six species. Grape contains high contents of anthocyanins and 5-methyltetrahydrofolate. Moreover, our data show that mango is rich in vitamin C and vitamin E, which resembles a previous work [39]. Furthermore, we have found that the contents of most vitamins and amino acids in mango are the highest among the three fruits, while the content of vitamin B6 in banana was the highest. Compared with the three fruits, the content of lipid in the three crops was higher. Although staple crops provide energy for humans worldwide, they lack essential nutrients. Although sweet corn contains relatively high levels of most amino acids and vitamins, the content of vitamins and amino acids in rice and wheat were relatively low. Rice mainly contained vitamin B1, and wheat mainly contained vitamin B6 and tryptophan. Our work discovered the complementary pattern of essential nutrients in crops and fruits.

Bioactive polyphenols from fruits have significant antioxidant activity [40]. For instance, catechin protects us against several diseases induced by oxidative stress, such as cardiovascular disease and cancer [41]. However, the direct application of catechins in food was limited by the low stability and content [42]. In this study, we draw the diversity of catechin in fruits, which provides insights into food choice based on the abundance of health-beneficial compounds. In addition, we also detected resveratrol in grape. It has strong antioxidant properties and the effects of protecting the heart and blood vessels, anti-arrhythmia, and vasodilation [43]. We found 11 specific metabolites in crops, mainly from flavonoids and lipids. These specific metabolites enriched the diversity of metabolites in different species and met humans` needs for nutrients.

## 5. Conclusions

This work identified the metabolic diversity in three major staple crops and three fruits, revealed the complementary patterns of nutrient accumulation of different species, and decoded the species-specific patterns of bioactive compounds. Among the three crops, sweet corn was rich in vitamins and amino acids, while rice and wheat were deficient in vitamins and amino acids. Among the three fruits, mango was rich in vitamins and amino acids. Compared with fruits, crops were rich in lipids. Overall, this work provided metabolomic evidence for a healthy diet, which aims to highlight the need for macronutrients and essential micronutrients [44].

## Figures and Tables

**Table 1:** Specific metabolites found in wheat, grape, and mango.

Species	ID	Q1 (Da)	RT (min)	Compounds	Class
Crops	hsf030	301.1	5.63	Diosmetin	Flavonoid
	MP0307	301.05	8.04	Chrysoeriol	Flavonoid
	MP0566	565.1	5.64	C-pentosyl-apigenin O-hexoside	Flavonoid
	MP0577	581.1	4.98	C-pentosyl-luteolin O-hexoside	Flavonoid
	hsl047	325.3	12.49	Heneicosanoic acid (C21:0)	Lipids
	MP0557	548.35	10.8	LysoPC 20:2	Lipids
	MP2054	307.3	11.39	cis-11,14,17-Eicosatrienoic Acid (C20:3)	Lipids
	hsb025	166.04	3.46	2-(Formylamino)benzoic acid	Others
	MP0885	287	12.14	5-deoxo-ent-10-oxodepressin	Others
	hsa126	161.2	0.72	D-Alanyl-D-Alanine	Amino acid and its derivatives
	MP0576	580.9	5.18	inositol pentakisphosphate	Organic acid and its derivates
Fruits	hsc303	307	3.5	(-)-Epigallocatechin	Flavonoid
	hsc317	291.08	3.58	Catechin	Flavonoid
	hsc325	307	2.57	(+)-Gallocatechin	Flavonoid
	hsc327	307	2.12	(-)-gallatechin	Flavonoid
	hsf421	451	3.72	Eriodictyol 7-O-glucoside	Flavonoid

## Data Availability

All data and materials are available on request.

## Supplementary Materials

The following supporting information can be downloaded at: https://www.mdpi.com/article/10.3390/foods11040550/s1, Table S1: Metabolic signals in the three fruits and three crops were detected by LC-MS-based non-targeted; Table S2: Metabolite variation in the three fruits and three crops were detected by LC-MS-based non-targeted; Table S3:Metabolites in the three fruits and three crops were detected by LC-MS-based targeted; Table S4: Specific metabolites in the three fruits and three crops were detected by LC-MS-based targeted; Table S5: Metabolite variation in the three fruits and three crops were detected by LC-MS-based targeted. Figure S1: Q trap 6500^+^ LC-MS/MS was used to analyze metabolite changes in crops and fruits. (a) Volcanic map analysis of differentially accumulated metabolites in rice and banana. (b) Volcanic map analysis of differentially accumulated metabolites in corn and banana. (c) Volcanic map analysis of differentially accumulated metabolites in wheat and banana. (d) Volcanic map analysis of differentially accumulated metabolites in rice and mango. (e) Volcanic map analysis of differentially accumulated metabolites in corn and mango. (f) Volcanic map analysis of differentially accumulated metabolites in wheat and mango.
