# Supplementary material for: Metabolomic Analysis Reveals Nutritional Diversity among Three Staple Crops and Three Fruits
Source: Foods. 2022 Feb 15;11(4):550. doi: 10.3390/foods11040550 (PMC8870860; doi:10.3390/foods11040550)
Supplement: Supplementary file 1 [file foods-11-00550-s001.zip › Supplement Figures.pdf]

Supporting information

Cross-Species Comparison of Metabolomics to Decipher the Nutrients Diversity in Crops and Fruits

Yunxia Shi[1], Yanxiu Guo[1], Yuhui Wang[1], Mingyang Li[1], kang Li[1,2], Xianqing Liu[1,2], Chuanying Fang[1] and Jie Luo[1, 2*]

1 School of Tropical Crops, Hainan University, Haikou 570288, China
2 Sanya Nanfan Research Institute of Hainan University, Hainan Yazhou Bay Seed Laboratory, Sanya, 572025, China
*Corresponding: jie.luo@hainanu.edu.cn (J.L.)

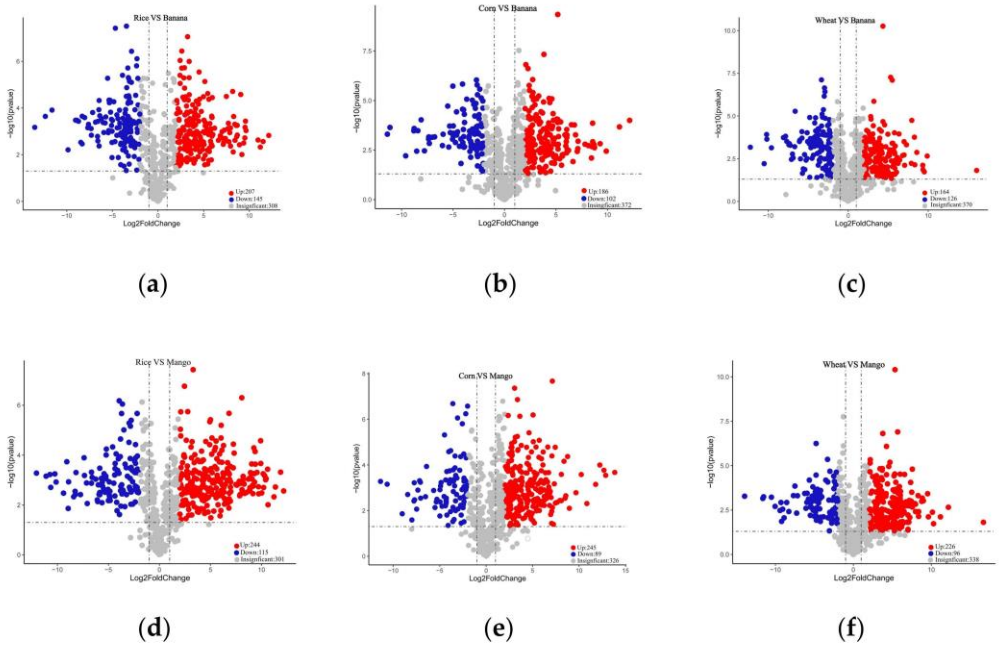

Figure S1. Q trap 6500+ LC-MS / MS was used to analyze the metabolites changes in crops and fruits. (a) Volcanic map analysis of metabolites differentially accumulated in rice and banana. (b) Volcanic map analysis of metabolites differentially accumulated in corn and banana. (c) Volcanic map analysis of metabolites differentially accumulated in wheat and banana. (d) Volcanic map analysis of metabolites differentially accumulated in rice and mango. (e) Volcanic map analysis of metabolites differentially accumulated in corn and mango. (f) Volcanic map analysis of metabolites differentially accumulated in wheat and mango.

Table S1. Metabolic signals in the three fruits and three crops were detected by LC-MS-based non-targeted (.xlsx)

Table S2. Metabolites variation in the three fruits and three crops were detected by LC-MS-based non-targeted (.xlsx)

Table S3. Metabolites in the three fruits and three crops were detected by LC-MS-based targeted (.xlsx)

Table S4. Specific metabolites in the three fruits and three crops were detected by LC-MS-based targeted (.xlsx)

Table S5. Metabolites variation in the three fruits and three crops were detected by LC-MS-based targeted(.xlsx)
